# Retrospective analysis of *Clostridium difficile* infection in patients with ulcerative colitis in a tertiary hospital in China

**DOI:** 10.1186/s12876-018-0920-x

**Published:** 2019-01-07

**Authors:** Hui Xu, Hao Tang, Tao Xu, Meng Xiao, Ji Li, Bei Tan, Hong Yang, Hong Lv, Yue Li, Jiaming Qian

**Affiliations:** 1 Department of Gastroenterology, Peking Union Medical College Hospital, Chinese Academy of Medical Sciences & Peking Union Medical College, Beijing, China; 2 Department of Epidemiology and Biostatistics, Institute of Basic Medical Sciences, Chinese Academy of Medical Sciences & School of Basic Medicine, Peking Union Medical College, Beijing, China; 3 Department of Clinical Laboratory, Peking Union Medical College Hospital, Chinese Academy of Medical Sciences & Peking Union Medical College, Beijing, China; 4 Beijing, 100730 China

**Keywords:** *Clostridium difficile*, Cytomegalovirus, Risk factor, Ulcerative colitis

## Abstract

**Background:**

Many reports have documented the increasing impact of *Clostridium difficile* infection (CDI) in patients with ulcerative colitis (UC). We conducted a retrospective study to determine the incidence, clinical characteristics, risk factors and prognosis of CDI in patients with UC.

**Methods:**

We studied patients with UC, hospitalized between January 2010 and December 2015 in a tertiary hospital in China. Stool samples were tested for *C. difficile* toxins A and B (CDAB) by enzyme immunoassays in UC patients with disease flare. CDI in UC patients was diagnosed by clinical symptoms and positive CDAB test, and each case was matched with CDAB-negative patients in a 1:2 ratio. Univariate and binary logistic regression analyses were used to measure the differences between patients with and without CDI.

**Results:**

Thirty-four (8.92%) of 381 patients with UC were CDAB positive. Antibiotic exposure within 3 months prior to the study (*P* = 0.004), hospitalization within 1 month prior to the study (*P* = 0.025), systemic use of steroids (*P* = 0.002) and active cytomegalovirus (CMV) infection (*P* = 0.001) were higher in CDI than non-CDI patients. Binary logistic regression analysis revealed that CMV infection was associated with CDI (odds ratio = 13.502, 95% confidence interval 1.307–139.512, *P* = 0.029). UC patients with *C. difficile* and CMV co-infection had more severe colonoscopic features.

**Conclusions:**

Recent use of antibiotics, prior hospitalization and systemic use of steroids increased the risk of CDI. CMV infection was an independent risk factor of CDI in patients with UC.

## Background

*Clostridium difficile* is a bacillus of gram-positive, spore-forming anaerobe, and was first identified in the 1970s. It was considered to be the main cause of pseudomembranous colitis [1]. *C. difficile* infection (CDI) has become a particular problem for patients with inflammatory bowel disease (IBD). Studies have found a significant increase in the incidence of CDI over the recent few decades. Patients with IBD have an increased risk of poorer outcomes when suffered from CDI, associated with higher frequency of flare-ups, greater morbidity and mortality, poorer response to treatment, higher rates of colectomy, need for more active treatment for IBD and longer duration of hospital stay [2, 3].

It is difficult to differentiate CDI in IBD patients from IBD flare, because the presentation is similar, which consists of abdominal pain and diarrhea. Many studies have tried to determine the potential risk factors of CDI in patients with IBD. Risk factors for CDI traditionally include age, antibiotic use, severe comorbidity or contact with hospital and other healthcare facilities [4]. IBD has been identified as an independent risk factor for CDI. Most patients of IBD with CDI have a history of IBD colitis (91%) [5]. Ulcerative colitis (UC) patients seem to account for the majority of CDI in the IBD population as a whole [6, 7].

The incidence of UC is higher than that of Crohn’s disease in China, [8, 9] while the epidemiology and risk factors of CDI in patients with UC are unclear in China. CDI has not been well acknowledged and routinely tested for in IBD patients with a flare-up. In our tertiary center, *C. difficile* testing of patients for an IBD flare has been gradually introduced as a routine procedure. This allowed us to conduct this retrospective study to assess the incidence and risk factors for CDI in patients with UC.

## Methods

### Patients

A retrospective, case–control, observational study was performed in patients with UC. The study population consisted of patients hospitalized from January 2010 to December 2015 in the Department of Gastroenterology of Peking Union Medical College Hospital; a tertiary hospital in Beijing, China. For patients with UC admitted for disease flare (with aggravative abdominal pain, bloody stool, increased bowel movements, with or without fever), stool samples were tested for *C. difficile* toxins A and B (CDAB). We recruited patients with confirmed diagnosis of UC, in accordance with the Concensus on Diagnosis and Treatment of Inflammatory Bowel Disease (2012), [10] and positive testing for *C. difficile* toxin. For each recruited case, two patients (controls), paired for gender, age and year of stool test, were randomly assigned from hospitalized patients with UC flare and negative *C. difficile* toxin test. This study was approved by the Institutional Committee of Science and Research Ethics (IRB number: S-K415).

### Demographic and clinical characteristics

Demographic characteristics included age, gender, smoking status and alcohol use. Clinical characteristics included the following. 1) Duration from UC diagnosis to CDAB testing, distribution of UC (Montreal classification), [11] UC severity (modified Truelove and Witts classification), UC endoscopic severity (modified Mayo score), [12] comorbid diabetes, history of colonic surgery, body mass index (BMI), complications (colon dilation), clinical manifestations (fever, abdominal pain, hematochezia, diarrhea defined by increased average bowel movements ≥3 times per day or increased water content in fecal compared with usual), peripheral venous white blood cell (WBC) count. 2) Potential risk factors: treatment of UC (5-aminosalicylic acid, systemic steroids, immunosuppressants, infliximab), recent (within 4 weeks) hospital admission, recent (within 3 months and 1 month) antibiotic exposure, recent (within 3 months) administration of proton pump inhibitors, recent (within 4 weeks) parenteral nutrition, presence of active cytomegalovirus (CMV) infection. We included not only patients with CMV colitis (diagnosed by tissue IHC), but also with CMV infection. Diagnosis of CMV infection was defined as the presence of any of the following in blood: positive CMV IgM, CMV DNA by polymerase chain reaction, CMV pp65. 3) Treatment and outcome: subsequent surgery, medication for CDI, outcome and recurrence of CDI.

### Evaluation of CDI

Diagnosis of CDI was based on a combination of clinical presence of diarrhea and laboratory findings. The patients enrolled all presented with diarrhea, and laboratory diagnosis of CDI was performed by detection of fecal CDAB through enzyme immunoassays (EIAs). Fecal samples were collected and sent to the Laboratory of Clinical Microbiology and stored at 2–8 °C, and then prepared for testing with the VIDAS *C. difficile* Toxin A&B Assay (BioMerieux, Marcy l’Etoile, France) [13]. Toxin concentrations were tested as relative fluorescence values (RFV) and the results were reported in terms of negative, equivalent and positive.

### Statistical analysis

The data were processed using SPSS version 21.0 (Chicago, IL, USA). The quantitative variables are expressed as mean and standard deviation or median and interquartile range. The qualitative variables are expressed as numbers and percentages. Univariate analysis was performed for continuous variables using a t-test or Wilcoxon rank-sum test. A chi-square test or Fisher’s exact test was used to reveal the differences between CDAB-positive and -negative patients. *P* < 0.05 was considered statistically significant. The variables with *P* < 0.05 in univariate analysis were entered in a binary logistic regression analysis.

## Results

### Demographic and clinical characteristics

A total of 381 patients with active UC were admitted from January 2010 to December 2015 and were tested for CDAB. Thirty-four of these were CDAB positive: 52.9% female, 47.1% male, and mean age 44.5 ± 15.5 years. Detected incidence rate of CDI in UC flares was 8.92% (34/381). Sixty-eight CDAB-negative patients with UC were matched as controls. Table 1 shows the demographic and clinical characteristics of these patients. There were no significant differences between the cases and controls for diabetes comorbidity (*P* = 1.000), smoking (*P* = 0.866), alcohol consumption (*P* = 0.218), duration of UC (*P* = 0.467), distribution of UC (*P* = 0.811), clinical severity of UC (*P* = 0.517), endoscopic score (*P* = 0.622), history of surgery (*P* = 1.000), clinical symptoms and colon dilation (*P* = 0.990). Most of the patients had extensive colitis, which was observed in cases (73.5%) and controls (75.0%). The mean number of bowel movements was 7.0 in both groups (*P* = 0.947).

**Table 1:** Demographic and Clinical Characteristics for Patients with UC

	*C. difficile* Positive *N* = 34		*C. difficile* Negative *N* = 68		
Factors	N	Median (P25, P75)	N	Median (P25, P75)	*P* value
Age (y)	34	44.5 (29.0, 60.0)	68	44.5 (29.0, 60.0)	1.000
Median duration from UC diagnosis to CDAB test (mo)	34	27.0 (7.0, 108.5)	68	36.0 (13.5, 117.0)	0.467
Number of bowel movements (times/d)	34	7.0 (4.5, 10.0)	68	7.0 (5.0, 10.0)	0.947
Factors	N	Mean ± SD	N	Mean ± SD	P value
BMI	34	20.4745 ± 3.82787	68	21.2466 ± 4.10828	0.390
Peripheral venous white blood cell (10^9^/L)	34	8.0676 ± 2.82308	68	8.0554 ± 2.95580	0.984
Peripheral neutrophils (10^9^/L)	34	5.9118 ± 2.82888	68	5.5834 ± 2.70716	0.571
Factors	N	Positive (%)	N	Negative (%)	
Male gender	16	47.1	32	47.1	1.000
Smoking status					0.866
Cessation ≥6 mo	2	5.9	7	10.3	
Active smoker	4	11.8	9	13.2	
Alcohol use					0.218
Cessation ≥6 mo	3	8.8	6	8.8	
Active use	4	11.8	2	2.9	
Diabetes	3	8.8	7	10.3	1.000
UC Truelove and Witts criteria					0.517
Mild	3	8.8	14	20.6	
Moderate	18	52.9	28	41.2	
Severe	13	38.2	26	38.2	
Mayo score at colonoscopy					0.622
1	5	15.6	8	12.3	
2	10	31.3	28	43.1	
3	17	53.1	29	44.6	
Distribution of UC					0.811
Proctitis	1	2.9	0	0.0	
Left-sided colitis	8	23.5	17	25.0	
Extensive colitis	25	73.5	51	75.0	
History of surgery	2	5.9	3	4.4	1.000
Symptom-fever	7	20.6	15	22.1	0.865
Symptom-abdominal pain	16	47.1	43	63.2	0.119
Symptom-bleeding	27	79.4	63	92.6	0.099
Symptom-diarrhea	29	85.3	65	95.6	0.113
Dilation of colon					0.990
No	33	97.1	66	97.1	
≥6 cm	1	2.9	1	1.5	
≥8 cm	0	0.0	1	1.5	
Subsequent surgery	9	27.3	11	16.2	0.189

*UC* ulcerative colitis, *CDAB Clostridium difficile* toxins A and B, *SD* standard deviation

### Risk factors for CDI

The risk factors for CDI were evaluated by univariate analysis (Table 2). Univariate analyses revealed systemic use of steroids (*P* = 0.002) increased the risk for CDI. The use of 5-aminosalicylic acid (5-ASA) was comparable between patients with CDI (85%) and without CDI (86%). Immunosuppressive treatment was not used commonly, such as anti-tumor necrosis factor agent infliximab.

**Table 2:** Possible Risk Factors For CDI in Patients with UC

Univariate Analysis					
	*C. difficile* Positive N = 34		*C. difficile* Negative N = 68		
Factors	N	Positive (%)	N	Negative (%)	P value
Use of 5-ASA	29	85.3	59	86.8	1.000
Use of systemic steroids	25	73.5	28	41.2	0.002
Use of immunosuppressants	5	14.7	5	7.4	0.610
Use of infliximab	1	2.9	2	2.9	1.000
Antibiotic exposure (within 3 mo)					0.004
No	18	62.1	52	88.1	
Yes	11	37.9	7	11.9	
Missing	5		9		
Antibiotic exposure (within 1 mo)					0.936
No	18	62.1	34	63.0	
Yes	11	37.9	20	37.0	
Missing	5		14		
Use of PPI (within 3 mo)					0.064
No	20	74.1	40	93.0	
Yes	7	25.9	3	7.0	
Missing	7		25		
Parenteral nutrition (within 1 mo)					0.134
No	15	60.0	42	76.4	
Yes	10	40.0	13	23.6	
Missing	9		13		
Prior hospitalization (within 1 mo)	23	67.6	30	44.1	0.025
Active CMV infection	14	42.4	10	14.7	0.001
Factors	N	Median (P25, P75)	N	Median (P25, P75)	P value
Dose of steroids (mg/d)	34	40.0 (0.0, 50.0)	68	0.0 (0.0, 30.0)	0.001
Multivariate Analysis – Logistic Regression					
Factor	Reference		OR (95% CI)		P value
Use of systemic steroids	Yes vs. No		2.059 (0.155–27.351)		0.584
Dose of steroids (mg/d)			0.990 (0.924–1.061)		0.769
Antibiotic exposure (within 3 mo)	Yes vs. No		10.246 (0.867–121.127)		0.065
Prior hospitalization (within 1 mo)	Yes vs. No		2.888 (0.528–15.802)		0.221
Active CMV infection	Yes vs. No		13.502 (1.307–139.512)		0.029

*UC* ulcerative colitis, *CDI Clostridium difficile* infection, *5-ASA* 5- aminosalicylic acid, *PPI* proton pump inhibitor, *CMV* cytomegalovirus.

Among CDI patients, 11 (37.9%) had antibiotic exposure within 3 months prior to admission, versus 7 (11.9%) patients in the control group (*P* = 0.004). There was no significant difference according to antibiotic exposure within 1 month prior to admission (*P* = 0.936). Twenty-three cases (67.6%) had been hospitalized during the previous month, compared to 30 of the controls (44.1%) (*P* = 0.025). CMV infection was more common in patients with CDI (14/34, 42.4%) than patients without CDI (10/68, 14.7%, *P* = 0.001). Logistic regression analysis revealed that the presence of active CMV infection was associated with CDI in patients with UC [odds ratio (OR) = 13.502, 95% confidence interval (CI): 1.307–139.512, *P* = 0.029].

**Fig. 1:**
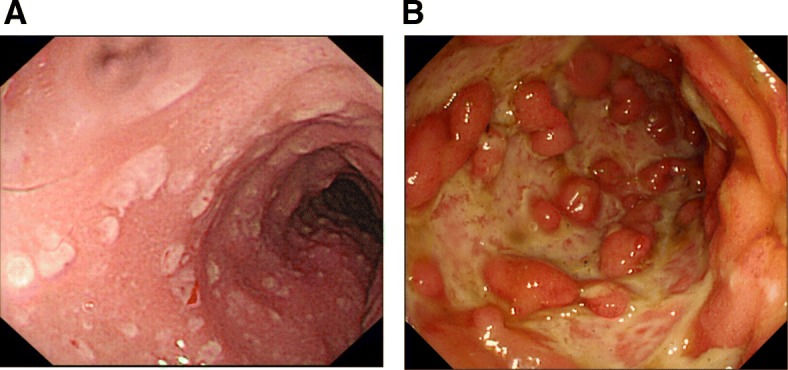
Colonoscopy appearance of patients with UC and CDI. (A) Pseudo-membrane was found in this patient with UC and CDI. (B) Severe colonoscopic appearance of a patient with UC and combined with CDI and CMV

Thirty-two of 34 patients with CDI had a colonoscopy 1 week before or after the CDAB test. Pseudo-membranes were found in only 2 (6.3%) patients (Fig. 1). Based on modified Mayo score, statistical analysis revealed that patients with *C. difficile* and CMV co-infection had a more severe appearance in colonoscopy than patients without CMV (the percentages of patients scoring 1, 2 and 3 were respectively 23.5, 53.0 and 23.5% in the non-CMV group, vs 7.7, 7.7 and 84.6% in the CMV group, *P* = 0.003).

### Treatment and outcome

During follow-up (from CDI to August 2016), 9 cases (27.3%) and 11 controls (16.2%) underwent ostomy or colorectomy (*P* = 0.189). None of the 34 patients with CDI died during follow-up. Twenty-five of 34 cases were treated for CDI at the physicians’ discretion if they considered that clinical exacerbation was related to CDI, and the median treatment course was 14 (IQR 11–17) days. 18 of the 25 patients were treated with metronidazole, 5 with vancomycin, and 2 with both vancomycin and metronidazole. 22 of the 25 cases had symptoms resolved, but it is difficult to distinguish whether the improvement of symptoms is the efficacy of treating CDI or the efficacy of treating UC combined with other anaerobic infections. The 9 patients who weren’t treated for CDI were treated for co-infected CMV, or enhanced treatment for UC of adding AZA or increasing dose of GCS, others had a repeated CDAB test and showed a negative result. All 9 patients had improved in clinical manifestation, and had a negative result of CDAB when repeated later. None of them underwent surgery later. There were 2 patients with recurrent CDI; both of who were treated with oral metronidazole and had concomitant CMV infection when first infected with *C. difficile*. One patient had recurrent CDI after 4.5 months, and then underwent total colectomy with ileostomy. The other patient had recurrent CDI after 1 month, and was administered cyclosporine for active UC and oral metronidazole again for CDI, and their diarrhea was resolved.

## Discussion

Patients with UC are at increased risk of acquiring CDI. The incidence of CDI with UC patients has also been rising in recent years [5, 7]. CDI mimics and exacerbates UC flare, therefore, it is essential that clinicians be alert to identify CDI and its risk factors, as treatment with corticosteroids without appropriate antibiotics may lead to deterioration [14]. The present study was a retrospective case–control study to determine the incidence rate and risk factors for CDI in patients with UC flare in a tertiary IBD center in China. We found that CDI occurred in 8.92% of patients with active UC who were hospitalized. CMV infection was associated with CDI in patients with UC according to univariate and binary logistic regression analyses. Recent antibiotic exposure (within 3 months), hospitalization (within 1 month) and systemic use of steroids were associated with CDI according to univariate analysis.

Studies from Europe and North America have shown that the incidence of CDI lies between 2.8 and 5.12% among patients with UC [3]. In China, however, the incidences of CDI in UC patients were reported 19.3 and 7.1% respectively [15, 16]. The difference in incidence among the studies may be due to the diversity of disease severity, sample size, and testing methods for *C. difficile*. In this study, the result was not really incidence in UC patients in general, it was incidence rate detected in active UC patients who was hospitalized in our tertiary hospital. Whether there is a real difference in the incidence of CDI between Asian and Caucasian populations needs further research. The demographic and clinical characteristics factors did not differ between the case and control groups in our study. The results reflect the difficulty in differentiation of CDI from UC flare clinically, and this is a challenge of clinical care, which reminds clinicians to screen for *C. difficile* at every flare in patients with UC.

It has been reported that risk factors for CDI in patients with UC are different from those of non-UC patients. Advanced age has been considered as a risk factor for CDI, [7] while in our study, age and gender, along with the year of stool testing were defined as matched factors and were excluded from the analysis, which could also have excluded relevant confounders. Most patients without IBD (53%) had nosocomial CDI, [17] while it is reported that most cases of CDI in IBD patients appear in the community [18, 19]. In the present study, 67.6% of the cases had been admitted during the previous months versus 44.1% of the controls, so hospitalization may still be a risk factor for CDI in patients with UC. The most important risk factor for CDI is antibiotic therapy in the general population. Hensgens et al. found that antibiotic use increased the risk for CDI during therapy and in the 3 months after cessation of antibiotic therapy. The highest risk for CDI was found during the first month after antibiotic use [20]. However, some researchers have reported that antibiotic use prior to admission is not an independent risk factor for CDI in patients with IBD [21, 22]. In this study, we found a significant increase in CDI within 3 months after cessation of antibiotic use, while antibiotic exposure within 1 month prior to admission did not increase the risk for CDI. While this result was come from the univariate analyses that might have confounding factors, multivariate analysis didn’t reveal that antibiotic exposure was associated with CDI, which was consistent with some literatures. At the same time, the retrospective nature of the research may cause recall bias because of missing of some data in medical record.

Glucocorticoids and immunomodulators may also be independent risk factors in patients with CDI [23]. In a large population-based cohort of IBD patients, corticosteroids were associated with a 3-fold increase in the risk of CDI compared with other immunomodulator or biological agents, irrespective of dose and duration [24]. Maintenance immunomodulators use is independently associated with CDI in IBD [5]. In this study, we found that systemic use of steroids was risk factor for CDI. Most patients in this study were treated with mesalamine, while immunosuppressive and anti-tumor necrosis factor agents were used in only a few patients, and their role in CDI needs further research.

Our study showed a definite correlation between active CMV infection and CDI in UC patients. CMV colitis has usually been reported co-infected with *C. difficile* among immunocompromised patients, who have human immunodeficiency virus infection, organ transplantation, malignant diseases, or IBD especially when receiving immunosuppressive agents [25]. CMV colitis can mimic pseudomembranous colitis in patients with immunocompromise status [25]. A history of CMV infection is reported as a significant risk factor for *C. difficile* infection [26]. Cases of concomitant CDI and CMV colitis in immunocompetent patients have also been reported. Coexisting CMV and *C. difficile* colitis may be refractory to first-line antibiotics for CDI treatment [27, 28]. In the present study, *C. difficile* and CMV co-infection had more severe colonoscopic features than CDI alone had. 2 cases of CDI recurrence were both co-infected with CMV. Co-infection with CMV may worsen CDI and influence the outcome of CDI in patients with UC.

It is reported that the recurrence rates of CDI were similar in patients treated with metronidazol and vancomycin [29]. However, 2 patients with recurrent CDI in this study were both con-infected with CMV, which is more serious clinically, and were both treated by metronidazol for CDI when infected the first time. It is reported that severe CDI may be more likely to cause recurrence, [30] and for severe CDI, vancomycin should be used as a first line [29]. Our research is a retrospective study, and more reliable conclusions should be clarified by further prospective studies.

Advances in medical management have led to a decreased colectomy rates in patients with UC in recent years, which was 16% within 10 years of diagnosis [31]. While CDI is associated with an increased risk of colectomy [32]. A meta-analysis reported that CDI was a significant risk factor for colectomy among patients with IBD, especially those with UC [33]. In the present study, although there was no significant difference between non-CDI and CDI groups, the rate of colectomy in the CDI group was higher than in the controls (27.3% vs 16.2%). The retrospective nature of our study and different follow-up periods may have caused recall bias.

There were several limitations to our study. First, testing methods for *C. difficile* mainly consist of nucleic acid amplifications tests (NAATs) for toxin genes or EIAs for toxins in the stools. We used an EIA for the diagnosis of CDI, which detects the presence of CDAB, and has low sensitivity compared to that of NAATs. It may have underestimated the rate of CDI in our UC patients. Second, this was a retrospective study, and not all patients admitted for active UC were tested for CDI especially before year 2012 when Chinese consensus on IBD management was issued, the exact incidence rate needs further research. Third, all patients enrolled were from a tertiary IBD center, who were patients with severe disease and the study results may not represent the overall CDI in Chinese UC patients. Fourth, though we thought to collect as much information as possible to observe the outcomes for CDI, the unequal follow-up window for patients may cause deviation. We considered the outcomes might be related to multiple factors such as UC activity and reaction to treatments. One additional limitation to this study is the lack of data about CMV detected on biopsies. On the other hand, we not only included patients with CMV colitis (diagnosed by tissue IHC), but also included patients with CMV viremia or reactivation (diagnosed by plasma DNA/pp65 or IgM) or so-called CMV infection. A prospective study and CDI test for all patients with UC flare are necessary to assess risk factors in these patients.

## Conclusions

In conclusion, we aimed to assess the incidence and risk factors for CDI in patients with UC in China. Detected incidence rate of CDI in UC flares was 8.92%. It is difficult to differentiate CDI in IBD patients from IBD flare clinically. Systemic steroids, antibiotic exposure and prior hospitalization may be risk factors for CDI in patients with UC. Active CMV infection has a noticeable correlation with CDI in UC patients. *C. difficile* and CMV co-infection may lead to worse endoscopic severity. CDI should be suspected and screened when exacerbation of UC occurs.

## Abbreviations

5-ASA: 5-aminosalicylic acid
CDAB: *C. difficile* toxins A and B
CDI: *Clostridium difficile* infection
CMV: Cytomegalovirus
EIAs: Enzyme immunoassays
IBD: Inflammatory bowel disease
NAATs: Nucleic acid amplifications tests
PPI: Proton pump inhibitor
RFV: Relative fluorescence values
UC: Ulcerative colitis
WBC: White blood cell
